# Where Are We to Be in These Times? The Place of Chronic Disease Prevention in Community Health Promotion

**Published:** 2007-06-15

**Authors:** Katherine M Wilson, Dawn W Satterfield

**Affiliations:** Division of Cancer Prevention and Control, National Center for Chronic Disease Prevention and Health Promotion, Centers for Disease Control and Prevention; Native Diabetes Wellness Program, Division of Diabetes Translation, National Center for Chronic Disease Prevention and Health Promotion, Centers for Disease Control and Prevention, Atlanta, Ga

In guiding public health strategists to promote community health at the Centers for Disease Control and Prevention's (CDC's) National Center for Chronic Disease Prevention and Health Promotion (NCCDPHP), the National Expert Panel on Community Health Promotion raised the knotty issue of the role of chronic disease prevention in community health promotion (1). The panel acknowledged the complexity of their recommendations to impact overall health — including the health of 46 million Americans who lack health insurance (2) — through multiple environments and policies. Nevertheless, panel members urged NCCDPHP and CDC to catalyze new relationships and expand its role as a standard-bearer and broker of public health practice to engage decision makers at local, state, national, and societal levels in creating healthier environments.

The authors' immediate response was to say that the health of the individual must be seen as linked to the health of the community more comprehensively than by focusing on a single chronic disease. Yet public health workers cannot abandon people who are ill from chronic diseases or conditions by reducing the research and public health practice that would help them improve. The authors concluded that the role of chronic disease prevention in community health promotion is as complex as the panel's recommendations. Through modifications in surveillance, intervention development and delivery, and collaboration with others, those of us in public health can work to integrate chronic disease prevention and care into community health promotion. From the vantage point of the field of chronic disease prevention and care, which is grounded in community and interconnectedness, we consider such integration to address the following question: Where are we to be in these times? Dorothy Nyswander's advice still rings clear and true, notes Caroline Wang — "where the people are" (3).

## From Risk Factor Epidemiology to Social Epidemiology

Beginning with the germ theory in the 19th century, a reliance on the ability of biomedicine to identify causes and treatments for illnesses charted the paths for early detection and treatment. Clinical trials and epidemiologic evidence on illness rates clarified the value of reducing individual risk factors by promoting early detection and treatment of cancer and complications of diabetes and heart disease, for example. While science has indeed helped tag, track, and address chronic illnesses and their risk factors, conventional epidemiologic approaches carry with them a danger of missing the social roots and distribution of illness (4). Risk-factor explanations for illness causation can be strong and predictive (e.g., obesity, physical inactivity), but they tend to perpetuate the idea that behavioral risks are determined solely by the individual rather than by a mix of individual and social factors (5). Examining disparities with a lens that includes social factors can provide richer explanations for epidemiologic rates. Epidemiologists at CDC's Division of Diabetes Translation have noted, for example, that cardiovascular disease (CVD) risk factors, except for diabetes, have generally decreased from 1971 to 2002. However, income- and education-related disparities in CVD risk factors persisted during the 3 decades, with little reduction in the past decade, and the increase in diabetes prevalence has primarily occurred among people with lower socioeconomic status (6).

Broadening the contributions to illness beyond individual risk factors to include sociological processes is encompassed within the domain of social epidemiology, first named in the 1950s (7). Social epidemiology is distinguished from conventional epidemiology by its insistence on investigating social determinants of population distributions of health, disease, and well-being, rather than by treating such determinants as mere background to biomedical phenomena (7). By collecting data on a few biological or behavioral factors, scientists limit exploration of the intricacy of contributing causes and solutions, which helps sustain a focus on the behaviors of individuals. The debates continue about which variables are important to context and how to measure them. Social determinants of health are societal conditions that affect health and can potentially be altered by social and health policies and programs (8). Pathways by which societal conditions affect health are being identified. Investigators in the CDC–National Institutes of Health-sponsored Translating Research Into Action for Diabetes (TRIAD) study conducted an extensive literature review to examine the relationship between socioeconomic position and the health of people with diabetes. This review revealed connections that informed the development of a conceptual framework to help identify interventions to reduce inequalities in health for people with chronic illnesses (9). The complexity of chronic disease causation, prevention, and care compels the linkage of numerous domains to unite social, biological, and statistical reasoning (10) to better understand the interactions of these phenomena. Because it is difficult to add questions to an established national surveillance system, linkages with other national datasets may provide an alternate method to extract information about the variables of interest. For example, linking data from the National Program of Cancer Registries with data from insurance claims would help us understand out-of-pocket costs for screening tests, which may tell us much about who obtains or might be able to obtain the tests, perhaps identifying new relationships among social determinants of health and specific chronic diseases and conditions. Even so, these data sets do not always help us find solutions.

## Building and Delivering Interventions 

The continued use of population-level data to identify individual risk rather than population risk encourages interventionists to focus on individuals at high risk while overlooking the risk-laden conditions that contributed to risk development in the first place (11). According to Smith et al, the word *research* is derived from the old French *reseachier*, which means "to look at again" (12). The time has come for interventionists to look more closely at context — the risk-laden conditions within communities — rather than limit the focus to individual risk factors, particularly as context relates to specific chronic diseases, such as cancer and diabetes.

Black women in the southeastern United States, for example, have a rate of having had a Papanicolaou (Pap) test in the last 3 years equal to or higher than white women in the same geographic area, but they have a higher rate of mortality from cervical cancer than white women (13,14). Individual factors such as age, heredity, and screening behavior do not fully explain the discrepancy. So what does? The quest for answers to this disease-specific issue led public health research back to the community to look again at the context in which decisions are made to obtain a Pap test. Public health researchers started asking questions about what women do to take care of themselves and to maintain their health and then focused on women's thoughts and feelings on cancer, cervical cancer, and receiving a Pap test. Listening and applying what was learned from the women will guide development of approaches that can improve the conditions and quality of local health care.

Another example of researching context and applying what is learned is provided by a study that is using focus groups composed of women of Mexican descent who had not had a Pap test in more than 4 years. The focus groups are designed to determine how the women view health and use the health care system. Intervention materials were developed not only for the women but also for the lay health workers, program managers, and administrators who worked with them. This approach attempts to create an intervention based on the views of the focus group participants.

The act of listening to community members also contributed to the development of the "Eagle Books" for young American Indian and Alaska Native children, a series of stories in which wise animal characters promote the gifts of healthy food and the joy of physical activity (15). The idea of using stories to relay important messages about health promotion and diabetes prevention arose from formative research (discussion groups with more than 300 tribal members) as well as from formal tribal consultation with the Tribal Leaders Diabetes Committee in collaboration with the Indian Health Service.

## Collaborations

Collaboration is not new to community health promotion, chronic disease, or public health. It is a powerful and efficient means of accomplishing objectives, enabling the creation of new allies and tackling cross-cutting issues. Programmatically, CDC's Division of Cancer Prevention and Control has moved away from a single-cancer focus by initiating and supporting the National Comprehensive Cancer Control Program (NCCCP) in 50 states, the District of Columbia, six territories, and six tribes or tribal organizations. The NCCCP pools resources to reduce the burden of cancer through risk reduction, early detection, better treatment, and enhanced survivorship (16).

A part of the next step for connecting cancer prevention and control with community health promotion was to link cancer control efforts with other chronic diseases. This linkage occurred when the regulation that reauthorized the National Breast and Cervical Cancer Early Detection Program allowed for the establishment of a program to educate women aged 40 to 64 with little or no health insurance about risk factors for other chronic diseases (17). All WISEWOMAN (Well-Integrated Screening and Evaluation for Women Across the Nation) projects provide women with blood pressure and cholesterol screenings (18). Some WISEWOMAN projects also offer tests for diabetes and osteoporosis, healthy-cooking classes, fitness competitions, or quit-smoking courses. The WISEWOMAN project has challenged grantees to be efficient and effective in offering screening for breast and cervical cancers and heart disease to the same women (18).

As public health researchers and communities collaborate, we must recognize the importance of self-governance within communities that choose to participate in identifying priorities and interventions. Communities with a strong sense of identity — a *unit of identity*, according to health education pioneer Guy Steuart — are well positioned to function as a *unit of solution* (19). For example, CDC awarded 3-year grants to eight tribes and tribal organizations in 2005 to identify practical environmental adaptations that support healthy choices and diabetes prevention and that complement other health and social justice programs in their communities. Interventions have included policy changes that affect community members across multiple generations, including school-menu and vending-machine options, communitywide health promotion messages, and the extension of walking trails (20).

The National Expert Panel on Community Health Promotion challenged CDC and NCCDPHP particularly to go one step further: to work across specific chronic diseases for the benefit of the community at large. Although future chronic disease interventions may incorporate many diseases, it is vital now to achieve widespread use of interventions that have been shown to be effective. An NCCDPHP workgroup has begun the cross-cutting task of building a framework for disseminating and implementing these proven interventions. The objectives of the workgroup are to achieve consistent communication between communities and multiple divisions within NCCDPHP, to improve the capacity of communities in identifying evidence-based interventions that fit their needs, and to encourage maximum leveraging of community resources throughout chronic disease activities.

## Conclusion

The authors believe that to respond adequately to the National Expert Panel on Community Health Promotion, the following should take place: data on social determinants of health should be collected along with conventional risk factors, interventionists should include the context of the community in trying to change individual health, and programmatic and research activities should become more interconnected within NCCDPHP. Linking methods of inquiry, knowledge, and action can seem daunting to public health agencies already strapped for resources and time. Yet the concept of interconnectedness has long described our work in public health. Wallack and Lawrence have proposed that the language of public health become more explicit about the interconnections to more clearly transmit our mission to the public (21).

Perhaps the ancient, holistic concept of interconnectedness and community was best expressed by Chief Seattle as he addressed the U.S. government in the 1850s during treaty negotiations for the Duwamish, Suquamish, and other Puget Sound tribes: "All things are connected like the blood that unites us all. Man does not weave the web of life; he is merely a strand in it. Whatever he does to the web, he does to himself" (22).
